# Prototype of an educational device for supporting and monitoring the treatment of type two diabetes mellitus

**DOI:** 10.1186/1758-5996-7-S1-A253

**Published:** 2015-11-11

**Authors:** Eduarda Mirela da Silva Montiel, Alessandro Murta Baldi, Ana Cláudia Garabeli Cavalli Kluthcovsky, Amaury A Castro

**Affiliations:** 1 Universidade Estadual de Ponta Grossa, Ponta Grossa, Brazil

## Background

Diabetes mellitus is considered an ongoing epidemic. Studies link poor adherence to treatment and encourage the development and implementation of technologies related to primary care of the disease.

## Objectives

To present the prototype of an educational and monitoring device to be used by patients with type two diabetes mellitus.

## Materials and methods

A prototype with four main components has been prepared: an Arduino Mega 2560 with a microcontroller board to prototype the device hardware and program components, an LCD display with integrated resistive touchscreen, used to make the interaction with the user, a buzzer tone generator for audible alert and AA batteries as a power source. Based on the Tamagotchi's method of use, toy which creates a virtual pet, a virtual pet has been created called “Togushi” for user interaction.

## Results

The Arduino development environment and programming language was used. Libraries that allow the date and time setting, alarm management, writing and drawing on the screen were installed. The prototype features a informing on diabetes mellitus and its treatment function, it also alerts the wright times for taking medication and registers the patient's personal characteristics (weight and height, for example). It features password security mechanisms and data encryption and a restricted area, via the web platform for access and monitoring by health professionals. It is an open source system, reusable, flexible and cost effective and can be adapted to other contexts and research in the health area.

## Conclusion

So far has not been found a similar educational device in Brazil for patients with the disease. The prototype is a great aid for the monitoring of patients with type 2 diabetes through health education.

**Figure 1:**
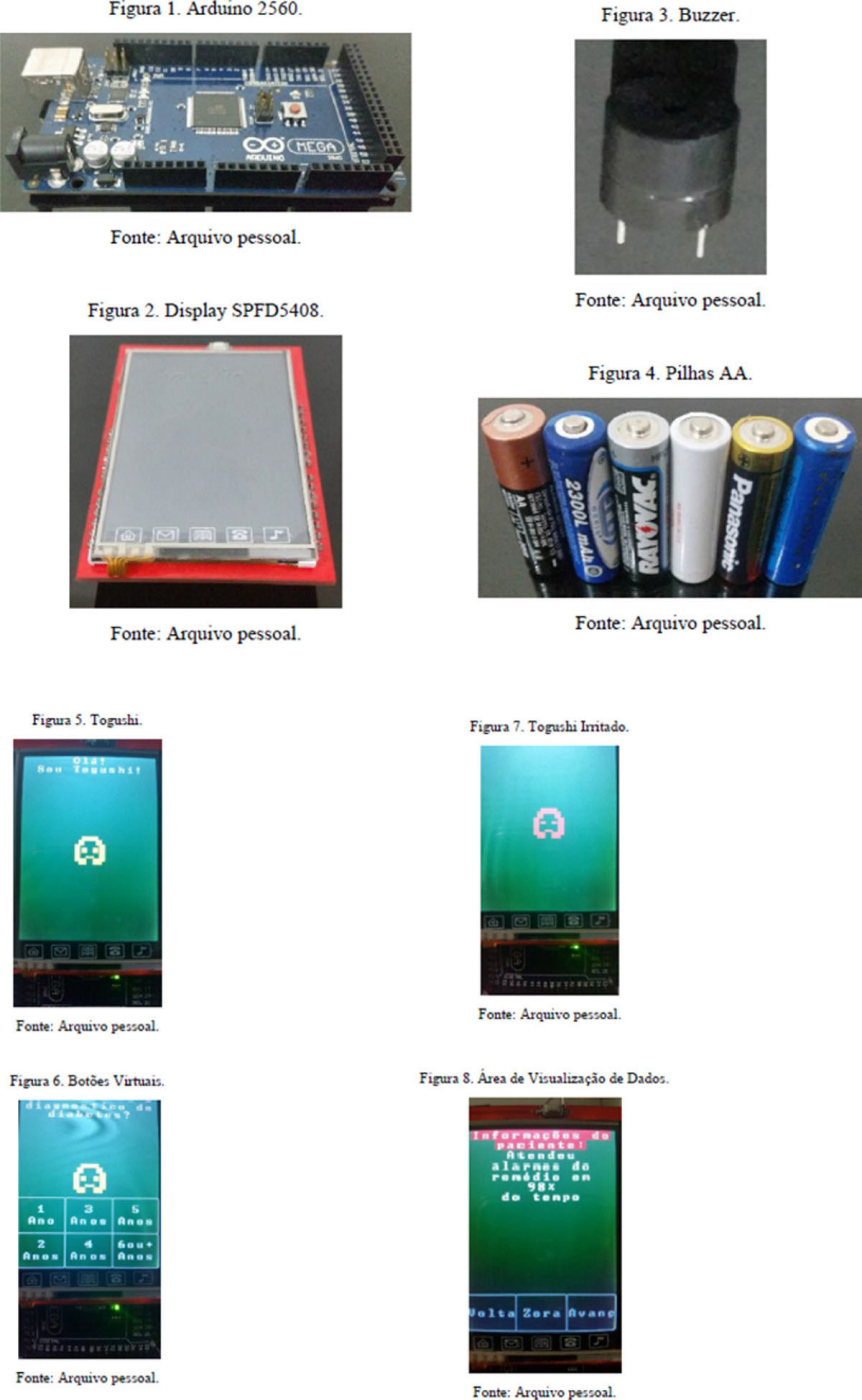
The prototype device.

